# Knowledge-Guided Symbolic Regression for Interpretable Camera Calibration

**DOI:** 10.3390/jimaging11110389

**Published:** 2025-11-02

**Authors:** Rui Pimentel de Figueiredo

**Affiliations:** Department of Mechanical and Production Engineering, Aarhus University, 8200 Aarhus, Denmark; rui@mpe.au.dk

**Keywords:** camera, calibration, distortion models, symbolic regression, genetic programming, model selection

## Abstract

Calibrating cameras accurately requires the identification of projection and distortion models that effectively account for lens-specific deviations. Conventional formulations, like the pinhole model or radial–tangential corrections, often struggle to represent the asymmetric and nonlinear distortions encountered in complex environments such as autonomous navigation, robotics, and immersive imaging. Although neural methods offer greater adaptability, they demand extensive training data, are computationally intensive, and often lack transparency. This work introduces a symbolic model discovery framework guided by physical knowledge, where symbolic regression and genetic programming (GP) are used in tandem to identify calibration models tailored to specific optical behaviors. The approach incorporates a broad class of known distortion models, including Brown–Conrady, Mei–Rives, Kannala–Brandt, and double-sphere, as modular components, while remaining extensible to any predefined or domain-specific formulation. Embedding these models directly into the symbolic search process constrains the solution space, enabling efficient parameter fitting and robust model selection without overfitting. Through empirical evaluation across a variety of lens types, including fisheye, omnidirectional, catadioptric, and traditional cameras, we show that our method produces results on par with or surpassing those of established calibration techniques. The outcome is a flexible, interpretable, and resource-efficient alternative suitable for deployment scenarios where calibration data are scarce or computational resources are constrained.

## 1. Introduction

Camera calibration is a foundational task in computer vision, robotics, and photogrammetry, where accurately modeling a camera’s intrinsic parameters directly impacts tasks such as 3D reconstruction, sensor fusion, and visual localization. A longstanding challenge in this area is selecting the most appropriate distortion model to account for the nonlinear and often asymmetric behavior of lenses, particularly wide-angle, fisheye, or catadioptric optics.

Traditional calibration techniques rely on the pinhole projection model combined with parametric distortion formulations such as the Brown–Conrady or radial–tangential model [1,2]. While effective in many settings, these models are limited in expressivity and may underperform when dealing with unconventional optics or distorted imaging environments. More expressive approaches, such as high-order polynomials [3], inverse distortion models [4], and deep neural networks [5], offer improved accuracy but introduce significant computational overhead, rely on large labeled datasets, and typically lack interpretability.

### 1.1. Motivation and Contributions

In this work, we introduce a symbolic regression framework for automatic model discovery in camera calibration. Our approach uses genetic programming (GP) to evolve symbolic expressions that describe lens distortion, while constraining the search using a grammar informed by domain knowledge. Specifically, the symbolic search space is populated not with generic mathematical operators alone but with physically meaningful model components, such as Brown–Conrady, Mei–Rives, Kannala–Brandt, and double-sphere, which have well-understood behaviors in optical systems. This grammar-guided design offers several advantages, particularly in maintaining interpretability despite the complexity of the discovered models. Each model component corresponds to a well-known distortion model with clear physical meaning. The modularity of the approach allows for the composition of more complex distortions while ensuring the transparency and traceability of each component. In particular, this design

1. restricts the solution space to interpretable, physically plausible models, reducing the risk of overfitting;
2. retains flexibility to discover new hybrid models by composing known distortions;
3. enables closed-form, differentiable expressions that can be refined via traditional nonlinear optimization (e.g., Levenberg–Marquardt [6]).

### 1.2. Summary of Contributions

This paper contributes the following:

1. A symbolic regression framework for intrinsic calibration, combining GP-based model discovery with domain-specific symbolic grammars;
2. An extensible model library incorporating classical and modern distortion formulations;
3. An empirical evaluation across diverse simulated lenses, demonstrating competitive or superior reprojection accuracy versus standard models.

By integrating physical priors into symbolic model discovery, our method offers a transparent, flexible, and computationally viable alternative to both rigid parametric and opaque black-box approaches.

To clearly isolate the potential contribution of symbolic regression for camera model discovery, we restrict our experiments to intrinsic calibration using synthetic and noiseless data. In order to ensure model generalization beyond the training data, we employ Monte Carlo cross-validation with a strict train/test split. This approach ensures that models are evaluated on unseen poses, mitigating the risk of overfitting. Additionally, model selection favors not only low reprojection errors but also parsimony by incorporating a complexity-penalized fitness function. This helps to prevent overly complex models that may only fit the training data well, improving their generalization across different pose sets. We assume a small set of known projection and distortion models and do not consider extrinsic parameters during model search. This setup avoids the added complexity and computational cost of full calibration and serves as an intermediate step toward a broader framework for the automatic discovery of novel, real-world distortion models, with finer primitive sets, ultimately composed of basic mathematical operations.

## 2. Related Work

Camera calibration plays a critical role in various fields. In robotics, it enhances simultaneous localization and mapping (SLAM), object manipulation, and sensor data fusion [7,8,9]. For autonomous vehicles, calibration supports tasks such as lane detection, obstacle recognition, and multicamera system alignment [10,11]. In augmented and virtual reality, it ensures accurate spatial alignment between virtual objects and the physical environment [12]. Additionally, in 3D reconstruction and photogrammetry, calibration is essential in producing precise metric models from images [13].

Recent developments include nonparametric and data-driven approaches like Gaussian process calibration [14] and deep learning-based camera modeling [5]. However, there is limited focus on methods that discover interpretable, equation-based models. This work advances the field by leveraging symbolic regression for automated model selection in camera calibration, providing a flexible and transparent alternative to both traditional parametric and black-box techniques.

Traditional calibration relies on the pinhole camera model combined with parametric distortion models such as the Brown–Conrady radial–tangential model [1,2,15]. While effective in many cases, these models often fail to capture complex distortions from fisheye or low-cost wide-angle lenses. More flexible models, including high-order polynomials [3], omnidirectional models [16], and inverse distortion approaches [4], offer improved accuracy but at the cost of increased complexity and sensitivity to data quality.

Deep learning-based methods [5] have also been introduced for distortion correction and model estimation. However, they typically require large datasets and lack interpretability, making them less suitable in data-scarce or safety-critical environments.

Selecting the appropriate distortion model is a critical yet often manual step in calibration workflows. To address this, symbolic regression and genetic programming (GP) have emerged as promising alternatives that automatically discover mathematical models from data [17,18]. Unlike predefined parametric models, symbolic regression can generate novel, interpretable expressions tailored to specific lens characteristics. Although computationally expensive during training, the resulting models are compact, efficient, and generalizable—making them well suited for offline calibration. While symbolic regression offers a powerful mechanism for discovering new analytical models that may better characterize specific lenses, in this work, we focus on model selection, leaving model discovery for future work.

## 3. Background

This section reviews foundational concepts for an understanding of camera projection models, coordinate systems, and lens distortion. We cover Cartesian and homogeneous coordinates in 2D and 3D, projective geometry’s role in camera modeling, the pinhole camera model, and lens distortion models accounting for real-world imaging imperfections.

### 3.1. Coordinate Systems

Coordinate systems assign numerical values, or coordinates, to uniquely specify points in space. Cartesian coordinates represent points in 2D as $\left(x,y\right)\in R^{2}$ and in 3D as $\left(X,Y,Z\right)\in R^{3}$. Homogeneous coordinates extend these to a projective space, enabling the representation of points at infinity and simplifying transformations. A 2D point (x,y) corresponds to homogeneous coordinates (x,y,w) with w≠0, convertible by

(1) $$\left(x,y\right)=\left(\frac{x}{w},\frac{y}{w}\right).$$

Similarly, a 3D point (X,Y,Z) becomes (X,Y,Z,w) with conversion

(2) $$\left(X,Y,Z\right)=\left(\frac{X}{w},\frac{Y}{w},\frac{Z}{w}\right).$$

This allows matrix multiplication to express affine and projective transformations.

### 3.2. Projective Geometry

Projective geometry studies properties that are invariant under projective transformations that preserve lines but not distances or angles, essential in modeling perspective projection from 3D to 2D.

#### 3.2.1. Pinhole Camera Model

A 3D point (X,Y,Z) projects to a 2D image point (u,v) through the camera center. First, the normalized image coordinates are

(3) $$x=\frac{X}{Z},\;y=\frac{Y}{Z},$$

representing projection onto a plane at Z=1. Pixel coordinates are then obtained via intrinsic parameters through a perspective transformation of the form

(4) $$u=f_{x}x+c_{x},\;v=f_{y}y+c_{y},$$

or, equivalently,

(5) $$\left[\begin{array}{c} u\\ v\\ 1 \end{array}\right]=K\left[\begin{array}{c} x\\ y\\ 1 \end{array}\right],\;K=\left[\begin{array}{ccc} f_{x} & 0 & c_{x}\\ 0 & f_{y} & c_{y}\\ 0 & 0 & 1 \end{array}\right].$$

Including extrinsic parameters (rotation R and translation t), the full projection is

(6) $$\left[\begin{array}{c} u\\ v\\ 1 \end{array}\right]=K\left[R|t\right]\left[\begin{array}{c} X\\ Y\\ Z\\ 1 \end{array}\right].$$

#### 3.2.2. Lens Camera Models

Real-world lenses introduce distortion that deviates from the ideal pinhole model. Lens distortion is modeled as a nonlinear function mapping the ideal (undistorted) image coordinates $x_{u}=\left(x_{u},y_{u}\right)$ to distorted coordinates $x_{d}=\left(x_{d},y_{d}\right)$:

(7) $$x_{d}=\phi \left(x_{u},d\right)$$

where ϕ is the distortion function, and d contains the distortion coefficients.

##### Brown–Conrady Model

The Brown–Conrady model [19] includes both radial and tangential distortion:

(8) $$\begin{array}{cc} D & =1+k_{1}r^{2}+k_{2}r^{4}+k_{3}r^{6},\;r=\sqrt{x_{u}^{2}+y_{u}^{2}},\\ x_{d} & =x_{u}D+2p_{1}x_{u}y_{u}+p_{2}\left(r^{2}+2x_{u}^{2}\right),\\ y_{d} & =y_{u}D+2p_{2}x_{u}y_{u}+p_{1}\left(r^{2}+2y_{u}^{2}\right). \end{array}$$

where *D* represents the radial distortion factor, *r* the radial distance from the center, and $k_{i}$, $p_{i}$ the radial and tangential distortion coefficients, respectively.

##### Rational Distortion Model (Extended Brown–Conrady)

The rational distortion model extends the classical Brown–Conrady formulation by introducing a rational (i.e., fractional) polynomial form for the radial distortion. This model is particularly effective in modeling complex lens distortions in wide-angle and consumer-grade cameras.

It includes six radial distortion coefficients $\left(k_{1},k_{2},k_{3},k_{4},k_{5},k_{6}\right)$ and two tangential coefficients $\left(p_{1},p_{2}\right)$. The distorted coordinates $\left(x_{d},y_{d}\right)$ are computed as

(9) $$\begin{array}{cc} x_{d} & =x_{u}\cdot \frac{1+k_{1}r^{2}+k_{2}r^{4}+k_{3}r^{6}}{1+k_{4}r^{2}+k_{5}r^{4}+k_{6}r^{6}}+2p_{1}x_{u}y_{u}+p_{2}\left(r^{2}+2x_{u}^{2}\right) \end{array}$$

(10) $$\begin{array}{cc} y_{d} & =y_{u}\cdot \frac{1+k_{1}r^{2}+k_{2}r^{4}+k_{3}r^{6}}{1+k_{4}r^{2}+k_{5}r^{4}+k_{6}r^{6}}+p_{1}\left(r^{2}+2y_{u}^{2}\right)+2p_{2}x_{u}y_{u} \end{array}$$

This rational formulation allows for high-accuracy distortion modeling by capturing both complex radial behavior and tangential effects.

##### Kannala–Brandt Model

Designed for fisheye lenses, the Kannala–Brandt model [20] models the radial distortion as

(11) $$r_{d}=\frac{r_{u}}{1+k_{1}r_{u}^{2}+k_{2}r_{u}^{4}+k_{3}r_{u}^{6}+k_{4}r_{u}^{8}}$$

The distorted coordinates are

(12) $$x_{d}=r_{d}\cdot \frac{x_{u}}{r_{u}},\;y_{d}=r_{d}\cdot \frac{y_{u}}{r_{u}}$$

where $r_{u}=\sqrt{x_{u}^{2}+y_{u}^{2}}$ is the radial distance in the undistorted image.

##### Mei–Rives Model

The Mei–Rives model [16] combines spherical projection with a pinhole camera model. A 3D point (X,Y,Z) is first projected onto the unit sphere:

(13) $$p_{s}=\frac{1}{\sqrt{X^{2}+Y^{2}+Z^{2}}}\left[\begin{array}{c} X\\ Y\\ Z \end{array}\right]$$

Then, it is projected to the image plane:

(14) $$p_{d}=\pi \left(p_{s}\right)=K\cdot \phi \left(p_{s}\right)$$

where K is the intrinsic camera matrix and $\phi (p_{s})$ accounts for lens distortion.

##### Equidistant Model

In the equidistant projection model, the image radius is proportional to the angle θ between the optical axis and the incoming ray:

(15) $$r=f\cdot \theta ,\;\theta =\arccos\left(\frac{Z}{\sqrt{X^{2}+Y^{2}+Z^{2}}}\right)$$

The corresponding image coordinates are

(16) $$x=r\cdot \frac{X}{\sqrt{X^{2}+Y^{2}}},\;y=r\cdot \frac{Y}{\sqrt{X^{2}+Y^{2}}}$$

where *r* is the radial distance from the center of the image.

##### Double-Sphere Model

The double-sphere model [21] projects a 3D point X=(X,Y,Z) onto two virtual spheres and then onto the image plane. Define the projection onto the unit sphere as

(17) $$p_{s}=\frac{X}{\sqrt{X^{2}+Y^{2}+Z^{2}}},\;\rho =\sqrt{p_{x}^{2}+p_{y}^{2}}$$

The distorted projection is then

(18) $$p_{d}=\frac{p_{s}}{\alpha \rho +\left(1-\alpha \right)\left(\xi +p_{z}\right)}$$

α∈[0,1] represents a blending factor that controls the transition between a pinhole-like and a fisheye-like projection, while ξ determines the distance between the two virtual spheres and thus the amount of distortion introduced by the model. Finally, the pixel coordinates are obtained as

(19) $$\left[\begin{array}{c} u\\ v \end{array}\right]=K\left[\begin{array}{c} p_{d}\\ 1 \end{array}\right]$$

This model is particularly useful in robotics and SLAM systems for calibration with wide-angle lenses.

## 4. Methodologies

Camera intrinsic calibration involves determining the internal parameters of a camera that govern how 3D points in the world are projected onto the 2D image plane. These intrinsic parameters are fixed properties of the camera, independent of its position or orientation, and include the focal length, principal point, and distortion coefficients.

The calibration process aims to minimize the *reprojection error*, the difference between the observed image points and the corresponding points projected by the estimated camera model. Formally, given a set of 3D object points,

(20) $$x_{i}={\left[X_{i},Y_{i},Z_{i}\right]}^{T},$$

and their corresponding 2D image points,

(21) $$p_{i}={\left[u_{i},v_{i}\right]}^{T},$$

the goal is to find camera parameters $P=[K,\phi \left(u_{u},d\right)]$, including intrinsic matrix K and distortion coefficients d, that minimize the sum of squared reprojection errors:

(22) $$E=\sum_{i=1}^{N}{∥p_{i}-f\left(x_{i},P\right)∥}^{2},$$

where $f(x_{i},P)$ is the projection function mapping 3D points to 2D image points considering distortion.

Expanding this error into pixel coordinates,

(23) $$E=\sum_{i=1}^{N}\left({\left(u_{i}-u_{d}\right)}^{2}+{\left(v_{i}-v_{d}\right)}^{2}\right),$$

where $\left(u_{d},v_{d}\right)$ are the distorted projected points depending on P. The calibration problem is thus

(24) $$P^{*}=\arg\min_{P}\sum_{i=1}^{N}\left({\left(u_{i}-u_{d}\left(P\right)\right)}^{2}+{\left(v_{i}-v_{d}\left(P\right)\right)}^{2}\right).$$

Optimization Methods

This nonlinear least-squares problem is commonly solved using the Levenberg–Marquardt algorithm [6], which iteratively updates parameters by

(25) $$P_{new}=P_{old}-{\left(J^{T}J+\lambda I\right)}^{-1}J^{T}e,$$

where J is the Jacobian matrix of residuals with respect to the parameters, λ is a damping factor, I is the identity matrix, and e is the vector of reprojection errors.

If the residuals are small, Gauss–Newton optimization can be used with updates

(26) $$P_{new}=P_{old}-{\left(J^{T}J\right)}^{-1}J^{T}e.$$

For multiview scenarios, *bundle adjustment* simultaneously optimizes the camera parameters and 3D points by minimizing the reprojection errors over all views, leading to more accurate calibration [22].

### 4.1. Planar Pattern-Based Calibration

Calibration using planar patterns (e.g., checkerboards) exploits the fact that all points lie on a flat surface, typically the plane Z=0. This reduces the problem to a 2D-to-2D mapping, simplifying the estimation of camera parameters.

Projection and Homography

A 3D point $x_{i}$ in the calibration pattern frame projects to image point $p_{i}$ as

(27) $$s\left[\begin{array}{c} u_{i}\\ v_{i}\\ 1 \end{array}\right]=K\cdot {\left[R|t\right]}^{v}\left[\begin{array}{c} X_{i}\\ Y_{i}\\ Z_{i}\\ 1 \end{array}\right],$$

where K is the intrinsic matrix, ${\left[R|t\right]}^{v}$ is the extrinsic transform for viewpoint *v*, and *s* is a scale factor.

Assuming that the pattern is planar ($Z_{i}=0$), this simplifies to

(28) $$\left[\begin{array}{c} u_{i}\\ v_{i}\\ 1 \end{array}\right]=K\left[\begin{array}{ccc} r_{1} & r_{2} & t \end{array}\right]\left[\begin{array}{c} X_{i}\\ Y_{i}\\ 1 \end{array}\right],$$

where $r_{1}$, $r_{2}$ are the first two columns of R.

This defines a homography $H_{v}$ between the planar pattern and the image:

(29) $$H_{v}=K\left[\begin{array}{ccc} r_{1} & r_{2} & t \end{array}\right].$$

Each view provides one homography satisfying

(30) $$\left[\begin{array}{c} u_{i}\\ v_{i}\\ 1 \end{array}\right]=H_{v}\left[\begin{array}{c} X_{i}\\ Y_{i}\\ 1 \end{array}\right].$$

The image coordinates explicitly are

(31) $$u_{i}=\frac{f_{x}\left(r_{11}^{v}X_{i}+r_{12}^{v}Y_{i}+t_{x}^{v}\right)}{r_{31}^{v}X_{i}+r_{32}^{v}Y_{i}+t_{z}^{v}}+c_{x}$$

(32) $$v_{i}=\frac{f_{y}\left(r_{21}^{v}X_{i}+r_{22}^{v}Y_{i}+t_{y}^{v}\right)}{r_{31}^{v}X_{i}+r_{32}^{v}Y_{i}+t_{z}^{v}}+c_{y}.$$

A single homography cannot uniquely separate intrinsic from extrinsic parameters, as changes in homography may stem from either. Hence, multiple views of the calibration pattern are necessary to resolve both intrinsic and extrinsic parameters by jointly solving multiple homographies.

This multiview approach ensures that intrinsic parameters are well constrained and the camera pose is accurately estimated.

### 4.2. Symbolic Camera Calibration

Camera calibration aims to find a model minimizing the reprojection error:

(33) $$E=\sum_{i=1}^{N}{∥p_{i}-f\left(x_{i},P\right)∥}^{2}$$

Traditional methods fix the model structure and estimate the parameters, while symbolic regression discovers both model forms and parameters [23,24]. By defining a set of operators and operands, symbolic regression flexibly searches for interpretable mathematical expressions that best fit the data.

**Figure 1:**
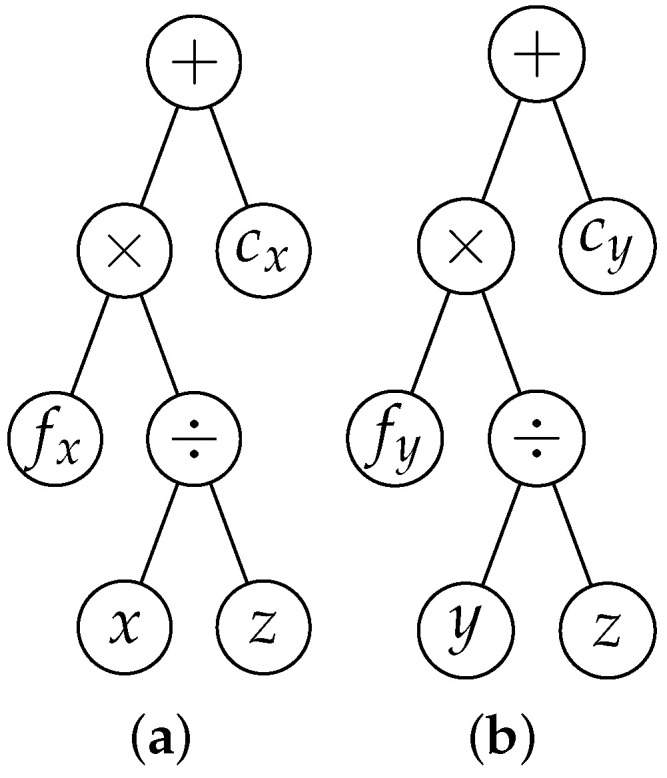
Expression trees representing the pinhole camera model, as defined in Equation (3).

Expressions are represented as hierarchical trees, with operators as internal nodes and variables/constants as leaves [25]. For example, the pinhole camera model (Equation (3)) can be represented as expression trees (Figure 1).

Symbolic regression solves

(34) $$\hat{f}=\arg\min_{f\in F}\sum_{i=1}^{N}{\left(y_{i}-f\left(x_{i1},\ldots ,x_{in}\right)\right)}^{2}$$

using evolutionary techniques like genetic programming to evolve models with improved accuracy and interpretability.

#### 4.2.1. Selecting Camera Models via Symbolic Regression

Given calibration data $D=\{\left(x_{i},p_{i}\right)\}$, the objective is to select and optimize a symbolic function $f(x_{i};\theta )$ that maps 3D points to 2D projections, by minimizing

(35) $$\sum_{i=1}^{N}{∥p_{i}-f\left(x_{i};\theta \right)∥}^{2},$$

where θ represents intrinsic and extrinsic parameters, as well as distortion coefficients.

##### Genetic Programming Setup

- Representation: Candidate solutions encode parameterized, predefined camera models (e.g., Brown–Conrady, Mei, Kannala–Brandt) as expression trees, combining fixed model structures with variables and constants.
- Initialization: The initial population $P\left(0\right)=\left\{I_{1},\ldots ,I_{N}\right\}$ consists of variants of these predefined models with randomized parameters.
- Fitness: Evaluated via the mean squared error (MSE) between the predicted projections and observed data.
- Selection: Employ roulette wheel or tournament selection to probabilistically favor fitter individuals.
- Genetic Operators:
  – Crossover: Exchange parameters or subtrees between parent models, preserving structural validity.
  – *Mutation*: Randomly perturb parameters or substitute subexpressions within models.
- Evolution: New generations are formed through elitism combined with genetic operators until convergence or stopping criteria are met.

#### 4.2.2. Parameter Optimization via Levenberg–Marquardt

Symbolic regression identifies model structures; parameters θ (e.g., focal lengths, distortion coefficients) are refined using the Levenberg–Marquardt algorithm [6]:

(36) $$\theta_{k+1}=\theta_{k}-{\left(J^{T}J+\lambda I\right)}^{-1}J^{T}r$$

where J is the Jacobian, *r* the residual, and λ the damping factor. This iterative update improves the parameter estimates and calibration accuracy.

## 5. Experiments

We evaluated our symbolic regression framework for camera calibration, implemented via the AlpineGP engine [26]. The aim is to learn compact symbolic expressions modeling projection and distortion across camera types. AlpineGP employs a grammar-guided evolutionary search with domain-specific primitives, well suited for geometric modeling. Performance is assessed via the reprojection error and runtime over various camera models, distortion profiles, and poses. Learned models are compared against established projection and distortion formulations from prior work.

### 5.1. Numerical Dataset Generation for Camera Calibration

**Figure 2:**
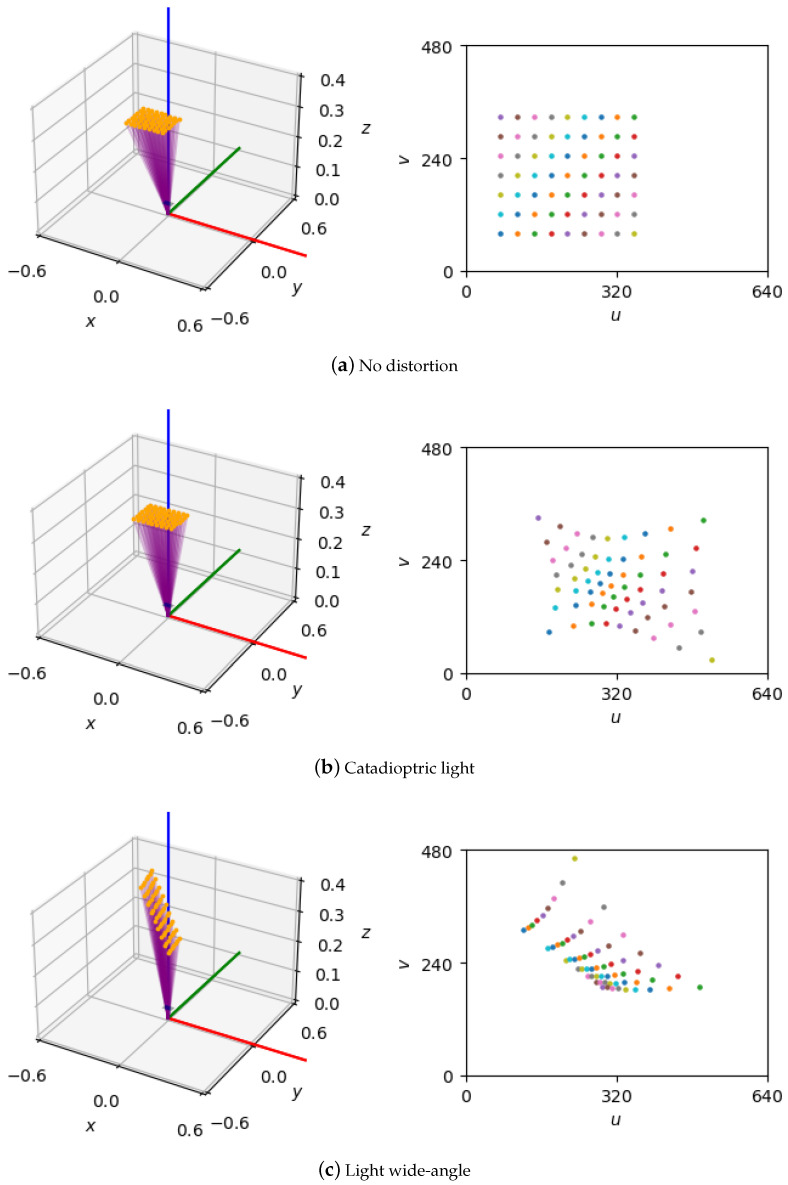
Sample calibration data: 9 × 7 coplanar points (**left**) and their image plane projections (**right**).

To evaluate calibration algorithms under controlled conditions, we generated a synthetic dataset of 3D points arranged in a planar chessboard pattern (7 rows, 9 columns), with 0.03 m corner spacing, spanning 0.24 m by 0.18 m. Each sample consists of 3D corner coordinates defined in the target’s local frame (Figure 2).

Pattern instances are randomly posed relative to a fixed camera frame, with translations uniformly sampled as x∈[−0.3,0.3] m, y∈[−0.2,0.2] m, z∈[0.01,0.35] m and rotations about each axis within ±25^∘^ (roll, pitch, yaw). A total of N=100 unique poses are generated to cover typical camera-to-pattern configurations.

The virtual camera has a resolution of 640 × 480 pixels, focal length of 35 mm, zero skew, a principal point at (320, 240), and an aspect ratio of 0.75, as summarized in Table 1.

Table 2 lists the distortion models included, covering a broad range of lens behaviors. This image-free dataset provides a controlled testbed for intrinsic and extrinsic calibration, enabling the isolated benchmarking of geometric estimation without image processing confounds.

### 5.2. Estimating the Search Space Size

Let *P* be the set of chosen primitives defined in Table 3. Each primitive f∈P has arity(f), i.e., the number of total arguments that it accepts. However, for distortion primitives (such as Brown–Conrady and Mei–Rives), only the first two or three arguments correspond to coordinate vectors and thus accept nested expressions, while the remaining arguments are fixed constants or parameters. Let $A_{f}$ represent the number of argument positions of each primitive where nested expressions are allowed, according to the following:

(37) $$A_{f}=\left\{\begin{array}{cc} 2\;\mathrm{or}\;3, & \mathrm{if}\;f\;\mathrm{is}\;a\;\mathrm{distortion}\;\mathrm{primitive};\\ \mathrm{arity}(f), & \mathrm{otherwise}. \end{array}\right.$$

We limit the expression depth to $D_{\max}$ and the total function nodes to $F_{\max}$. The total number of possible trees is

(38) Ntrees=∑k=1Fmax∑treeT|T|=k,depth(T)≤Dmax∏f∈TAf×NterminalsLT,≈∑k=1Fmax|P|k·Aavgk·Nterminalsk+1

where |T| is the total nodes in the tree, |P| is the size of the primitive set, depth(T) is the maximum depth of the tree, $N_{\mathrm{terminals}}$ is the number of terminal symbols (nonconstant arguments plus constants), $L_{T}$ is the count of leaves, and $A_{\mathrm{avg}}$ is the average of $A_{f}$ across all primitives.

For our set of |P|=11 primitives, we constrain the expression depth to $D_{\max}\;=\;3$ and the total function nodes to $F_{\max}=4$. Assuming conservatively that $A_{\mathrm{avg}}\approx 2$ and $N_{\mathrm{terminals}}\approx 8$ (3 coordinates plus 5 constant terminals), then

(39) Ntrees≳∑k=1411k·2k·8k+1.

This yields a coarse lower bound of $N_{\mathrm{trees}}∼10^{8}$, i.e., hundreds of millions of possible expression trees.

Symbolic regression over even this modest-sized space is infeasible via exhaustive search, particularly because each candidate expression often requires costly parameter fitting (e.g., via Levenberg–Marquardt optimization). Symbolic regression is formally known to be NP-hard [27], and the combinatorial explosion outlined above underscores why genetic programming remains one of the most practical and scalable strategies for exploring such high-dimensional expression spaces. In contrast, the brute-force enumeration of all possible nested compositions is computationally prohibitive, especially when accounting for both the nested argument structure and the per-candidate parameter optimization.

Taken together, for our use case, these considerations justify the need to (1) limit primitives to meaningful camera model functions, (2) bound the expression depth and function count to ensure tractability, and (3) employ genetic algorithms to efficiently explore the resulting combinatorial space.

On our test workstation (Intel i9 CPU, 32 GB RAM), each symbolic regression run takes approximately 15–20 min per model, depending on the complexity of the search space and parameter optimization. In comparison, traditional calibration methods (e.g., OpenCV) are typically completed in seconds. However, our method is intended for offline calibration scenarios, where interpretability and model discovery are prioritized over runtime.

### 5.3. Evaluating Calibration Performance

The symbolic regression genetic programming optimizer was configured with the parameters described in Table 4, aiming to balance exploration and exploitation within the search space for the efficient identification of symbolic expressions that accurately represented the underlying data. The hyperparameters in Table 4 were initially selected based on common genetic programming practice and refined through a limited grid search on validation tasks. We acknowledge that a systematic hyperparameter tuning process or sensitivity analysis would provide deeper insight into the robustness of the framework.

For evaluation, we employed Monte Carlo cross-validation [28], a technique involving the random subsampling of the dataset multiple times to reduce variance in the performance estimates, leading to more reliable evaluations. Each iteration involved splitting the dataset into training and testing subsets, training the model on the training subset, and evaluating it on the testing subset. The evaluation metrics from each iteration were then averaged to provide a more robust assessment of the calibration algorithm’s generalization capabilities. In our study, we performed 10 trials, each involving a random 30/70 train/test split. The choice of a 30/70 train/test split with 10 Monte Carlo trials was intended to evaluate generalization while retaining enough test data to measure robustness. We note that alternative validation schemes (e.g., k-fold cross-validation or leave-one-out) could have enabled more comprehensive assessments, especially given the small dataset size. However, k-fold methods would require repeated model discovery for each fold, substantially increasing the computational cost. The results from these trials were averaged to obtain a more stable estimate of the calibration algorithm’s effectiveness.

Reprojection Error

To assess the accuracy of the symbolic regression models, we use the reprojection error, a standard metric in camera calibration that measures the Euclidean distance between the observed 2D image points and their reprojected counterparts from 3D world coordinates:

(40) $$E_{\mathrm{reproj}}={\left|x_{\mathrm{observed}}-x_{\mathrm{projected}}\right|}_{2}$$

where $x_{\mathrm{observed}}$ and $x_{\mathrm{projected}}$ denote the true and predicted image coordinates, respectively [29].

**Figure 3:**
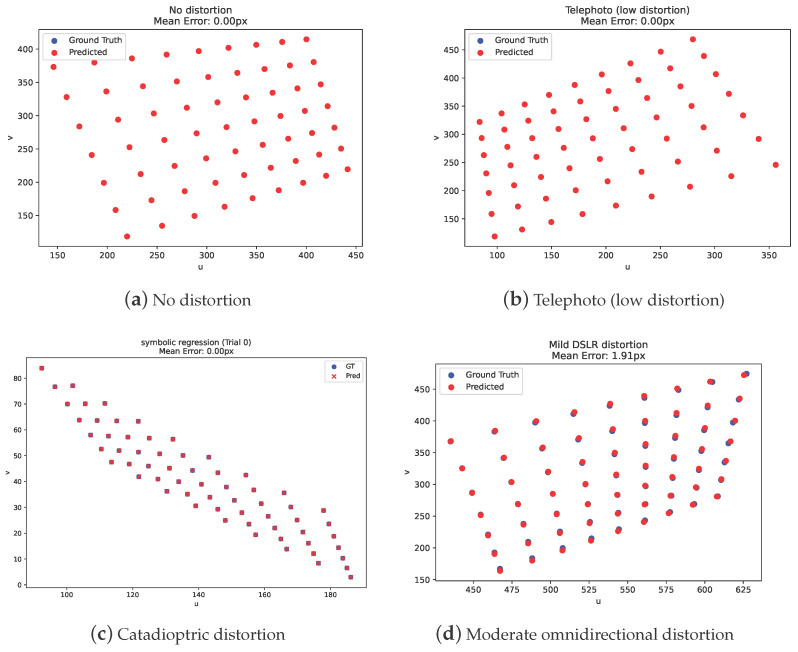
Example reprojections for the obtained symbolic regression models under different distortion profiles.

Table 5 reports the mean reprojection errors across various lens distortion profiles for our symbolic regression models alongside standard OpenCV calibration models (e.g., fisheye, pinhole, omnidir, rational). Figure 3 shows qualitative results on test images. The symbolic regression models consistently achieve low reprojection errors across the majority of camera types. In low-distortion settings (e.g., “no distortion” and “telephoto”), symbolic models reach subpixel accuracy, being comparable to or better than OpenCV’s best-fitting parametric models. For moderate distortions (e.g., “light fisheye,” “catadioptric light”), the symbolic models continue to outperform traditional models, with near-zero errors. Notably, as can ben seen in Table 6, two discovered models (light fisheye and light wide-angle) are not the ground truth projection functions yet achieve a perfect data fit with a zero reprojection error. This underscores symbolic regression’s ability to uncover structurally different but functionally equivalent models in complex projection spaces.

However, for highly nonlinear or extreme distortion profiles—such as those found in 360° cameras or extreme hyperbolic projections—the symbolic model exhibits greater variability and increased errors compared to OpenCV’s omnidirectional models, which are specifically designed to handle such complex geometries. This performance gap is partially attributed to the sensitivity of symbolic regression to internal constant optimization, particularly in deeper or more highly curved projection functions. Although extended genetic optimization times could mitigate this limitation, symbolic models remain competitive overall. In many scenarios, they outperform traditional rational and fisheye models—even in the absence of manual tuning or domain-specific constraints.

Overall, these results highlight symbolic regression as a viable and often superior alternative to traditional calibration models. Unlike OpenCV’s fixed-function distortions, our models are discovered automatically and can yield compact, interpretable expressions tailored to each distortion scenario. This opens the door for calibration systems that are both model-agnostic and semantically meaningful, especially in applications requiring generalization or analytical guarantees.

## 6. Conclusions

In this work, we present a symbolic regression framework for the automatic discovery of camera calibration models, using genetic programming to search over a space of interpretable geometric primitives. Our approach recovers compact, closed-form expressions that rival or outperform those of traditional parametric models in reprojection accuracy across a wide range of distortion profiles—including pinhole, fisheye, catadioptric, and omnidirectional lenses.

By producing models that retain the semantic structure, our method offers greater interpretability compared to black-box or high-degree polynomial models. An analysis of the symbolic search space reveals a combinatorially large number of possible expressions (on the order of $10^{8}$), justifying the use of heuristic search over brute-force methods.

Empirically, the symbolic models achieve near-zero reprojection errors in undistorted and mildly distorted settings and remain competitive in more complex cases. The performance in highly nonlinear regimes can be further improved by tightening the optimizer tolerances (e.g., lowering xtol, ftol, gtol), enabling finer parameter tuning at the cost of computation.

### Current Limitations and Future Work

While our initial experiments focused on synthetic datasets, this setup enables controlled comparisons, repeatability, and detailed analysis of the discovered models across diverse lens types. However, we acknowledge the importance of evaluating performance on real-world calibration tasks involving standard targets (e.g., checkerboards), various lens types, and challenges such as noise, blur, or illumination variability. These are key directions for future work.

Importantly, this study focused solely on intrinsic calibration, deliberately excluding the estimation of extrinsic parameters (i.e., rotation and translation between camera and world coordinates). While the joint optimization of intrinsics and extrinsics is standard in practical calibration pipelines, including extrinsics significantly increases the computational burden, especially when nested within a symbolic search loop. As such, we defer integrated intrinsic–extrinsic optimization to future work, where we will explore ways to manage this additional complexity efficiently.

The primitive set was chosen in our experiments based on prior work in camera calibration, including distortion models commonly used in the literature (e.g., Brown–Conrady, double-sphere, FOV). Our selection was guided by the goal of enabling expressive yet interpretable model discovery. We did experiment with alternative primitive sets, including expanded and reduced versions. Initial findings suggested that a larger primitive set would improve the expressiveness but increase the search time and risk of overfitting. Conversely, a minimal set may limit model discovery. A comprehensive ablation study on primitive selection is planned as part of our future work.

We further aim to refine model discovery by increasing the granularity of the symbolic primitives, enabling the system to build models from lower-level mathematical operations (e.g., powers, trigonometric terms). This may allow the emergence of novel, hybrid formulations that generalize beyond established models. Finally, we plan to investigate integrated structure-and-parameter optimization to improve the robustness and scalability for real-world deployment. 

## Figures and Tables

**Table 1:** Virtual camera intrinsics used for dataset generation.

Parameter	Value
Resolution	640 × 480 pixels
Focal length	35 mm
Skew	0
Principal point	(320, 240)
Aspect ratio	0.75

**Table 2:** Camera distortion profiles used in the dataset.

Label	Model	Coefficients
No distortion	Pinhole	[]
Telephoto (low distortion)	Brown–Conrady	[−0.01, 0.001, 0.0001, −0.0002, 0.0]
Light fisheye	Kannala–Brandt	[0.05, −0.01, 0.005, −0.001]
Catadioptric light	Mei–Rives	[0.5]
Moderate omnidirectional	Mei–Rives	[1.0]
360 camera	Mei–Rives	[1.5]
Extreme hyperbolic	Mei–Rives	[2.0]
Light wide-angle	Equidistant	[0.01, −0.005, 0.0, 0.0]

**Table 3:** Basic primitives used for camera modeling in symbolic regression.

Primitive Name	Description	Purpose in Camera Modeling	Arguments
normalize	Computes normalized image plane coordinates: $\frac{X}{Z}$ or $\frac{Y}{Z}$	Projects 3D points to 2D before applying distortion or intrinsics	X or Y, Z
linear_affine	Applies scale·x+offset	Models scaling and shifting (e.g., focal length, principal point)	x or y, scale, offset
brown_conrady	Classical Brown–Conrady radial–tangential model	Captures lens distortion using radial and tangential terms	x or y, y or x, k1, k2, p1, p2, k3
kannala_brandt	Odd-order polynomial fisheye model	Models extreme wide-angle distortions	x or y, y or x, k0, k1, k2, k3
mei_rives	Spherical projection with mirror parameter ξ	For central catadioptric (mirror-based) systems	X or Y, Y or X, Z, ξ
equidistant	Equidistant fisheye model	Ensures angle from optical axis maps linearly to radius	x or y, y or x, k1, k2, k3, k4
double_sphere	Two-sphere projection model with ξ, α	Models ultra-wide FOV more accurately than pinhole	X or Y, Y or X, Z, ξ, α
rational	Rational model: $\frac{P(x,y)}{Q(x,y)}$	Flexible model using polynomial numerator/denominator	x, y, k1–k6
omnidirectional	Polynomial mapping for omnicameras	Approximates wide-angle views with polynomial terms	x or y, y or x, c0–c3

**Table 4:** Experimental parameters for symbolic regression optimization.

Parameter	Value
Population Size ($N_{\mathrm{individuals}}$)	100
Generations ($N_{\mathrm{gen}}$)	100
Multi-Island Model ($N_{\mathrm{islands}}$)	10
Crossover Probability ($P_{\mathrm{crossover}}$)	0.7
Mutation Probability ($P_{\mathrm{mutation}}$)	0.3

**Table 5:** Reprojection errors (in pixels) for various distortion profiles and camera models.

Dataset	Camera Calibration Model		
	Pinhole	Rational	Symbolic Regression
No distortion	**0.000 ± 0.000**	**0.000 ± 0.000**	**0.000 ± 0.000**
Telephoto (low distortion)	**0.000 ± 0.000**	**0.000 ± 0.000**	**0.000 ± 0.000**
Light fisheye	0.716 ± 0.505	0.764 ± 0.536	**0.000 ± 0.000**
Catadioptric light	0.147 ± 0.091	0.125 ± 0.089	**0.000 ± 0.000**
Moderate omnidirectional	0.406 ± 0.331	0.376 ± 0.322	**0.286 ± 1.648**
360 camera	0.745 ± 0.487	**0.725 ± 0.484**	3.323 ± 4.915
Extreme hyperbolic	**0.358 ± 0.217**	0.644 ± 0.480	1.778 ± 2.611
Light wide-angle	0.291 ± 0.203	0.265 ± 0.175	**0.000 ± 0.000**

**Table 6:** Symbolic models with parameterized functions.

Dataset	Symbolic Model
No distortion (Best U)	$linear\_affine\left(normalize\left(x,z\right),320.0,320.0\right)$
No distortion (Best V)	$linear\_affine\left(normalize\left(y,z\right),359.19,240.0\right)$
Telephoto (low distortion) (Best U)	$linear\_affine\left(brown\_conrady\left(double\_sphere\left(x,y,z,0.2,-0.21\right),normalize\left(y,0.82\right),0.0,0.0,0.0,0.0,0.0\right),384.23,319.96\right)$
Telephoto (low distortion) (Best V)	$linear\_affine\left(brown\_conrady\left(normalize\left(y,z\right),normalize\left(x,z\right),-0.01,0.0,0.0,0.0,0.0\right),359.19,240.0\right)$
Light fisheye (Best U)	$linear\_affine\left(kannala\_brandt\left(double\_sphere\left(x,y,linear\_affine\left(z,5.22,0.0\right),-0.81,0.7\right),y,0.33,0.14,0.03,0.05\right),311.98,320.0\right)$
Light fisheye (Best V)	$linear\_affine\left(double\_sphere\left(kannala\_brandt\left(y,x,0.33,0.14,0.04,0.04\right),x,linear\_affine\left(z,5.21,0.0\right),-0.81,0.69\right),350.03,240.0\right)$
Catadioptric light (Best U)	$linear\_affine\left(mei\_rives\left(x,y,z,0.5\right),320.0,320.0\right)$
Catadioptric light (Best V)	$linear\_affine\left(mei\_rives\left(y,x,z,0.5\right),359.19,240.0\right)$
Moderate omnidirectional (Best U)	$linear\_affine\left(mei\_rives\left(x,y,z,1.0\right),320.0,320.0\right)$
Moderate omnidirectional (Best V)	$linear\_affine\left(mei\_rives\left(y,x,z,1.0\right),359.19,240.0\right)$
360 camera (Best U)	$linear\_affine\left(mei\_rives\left(x,y,z,1.5\right),320.0,320.0\right)$
360 camera (Best V)	$linear\_affine\left(mei\_rives\left(y,x,z,1.5\right),359.19,240.0\right)$
Extreme hyperbolic (Best U)	$linear\_affine\left(mei\_rives\left(x,y,z,2.0\right),320.0,320.0\right)$
Extreme hyperbolic (Best V)	$linear\_affine\left(mei\_rives\left(y,x,z,2.0\right),359.19,240.0\right)$
Light wide-angle (Best U)	$linear\_affine\left(equidistant\left(double\_sphere\left(x,y,linear\_affine\left(z,149.6,0.0\right),-0.99,0.65\right),y,0.33,0.15,0.02,0.06\right),318.35,320.0\right)$
Light wide-angle (Best V)	$linear\_affine\left(double\_sphere\left(y,x,linear\_affine\left(z,142.48,0.0\right),-0.99,0.65\right),357.37,240.0\right)$

## Data Availability

The original contributions presented in this study are included in the article. Further inquiries can be directed to the corresponding author.
